# Innovative Modifications in Diamond Umbilicoplasty: Simplified Techniques for Improved Cosmetic Results

**DOI:** 10.7759/cureus.75353

**Published:** 2024-12-09

**Authors:** Mohamed B Ahmed, Fatima Al-Mohannadi, Khaled E Elzawawi, Nasrin Jafarian, Sara Alharami, Shakir Al-Mashhadani, Abeer Alsherawi

**Affiliations:** 1 Plastic and Reconstructive Surgery, Hamad Medical Corporation, Doha, QAT; 2 College of Medicine, QU Health, Qatar University, Doha, QAT; 3 Medical Education, Hamad General Hospital, Hamad Medical Corporation, Doha, QAT

**Keywords:** abdominoplasty, aesthetic surgery, cosmetic scar, surgical techniques, umbilicoplasty

## Abstract

Umbilicoplasty is one of the main steps in abdominoplasty procedures. Various techniques of umbilicoplasty are performed by surgeons from diverse backgrounds, all aiming for a naturally appearing umbilicus with minimal scarring. In this report, we present two umbilicoplasty methods practiced at our center. The key steps involved in both techniques include securing the umbilicus in its new position, creating a natural umbilical depth, and preventing visible scarring. These two methods are easily reproducible, time-effective, and consistently yield aesthetically pleasing results. We also discuss alternative umbilicoplasty methods performed in other facilities.

## Introduction

The umbilicus is considered one of the major components of the abdominal wall. Fundamentally, it is a healed scar that forms as a consequence of cutting the umbilical cord at birth [1]. Although it is essentially a scar, it is regarded as an important aesthetic feature of abdominal appearance, and many cultures/individuals value a visually pleasing umbilicus. Several factors impact the visual aesthetic of the umbilicus, including its size, shape, depth, position, and location on the abdomen [2].

Umbilicoplasty is an essential step in various surgical procedures. For instance, abdominoplasty is one of the most common procedures that incorporates umbilicoplasty as a critical step [3]. There are several umbilicoplasty techniques including but not limited to the round incision technique, Borges Technique, C-V flap technique, modified “unfolded cylinder” technique, lunch box-type technique, V-Y flap technique, inverted V-Y flap technique, inverted U flap technique, twisted flaps technique, triangular flap technique, iris technique, double-C technique, and purse-string suture techniques [2,4]. The most commonly used umbilicoplasty technique is the round incision technique, which results in a round-shaped umbilicus [2]. Irrespective of the technique used in umbilicoplasty, the result should yield an outcome that is reliable, reproducible, aesthetically pleasing, and associated with a low risk of morbidity, infection, and necrosis [4].

In this article, we aim to compare two different umbilicoplasty techniques performed by two consultants at our center. We also engage in a discussion of the benefits of each method as well as a review of various other techniques published in the literature.

## Technical report

The abdominal incision and flap dissection are initiated according to the marked abdominoplasty lines until the umbilical region is reached. The umbilicus is then identified, and a precise incision is made in the skin, followed by careful dissection of the umbilical stalk down to the fascia to detach it from the abdominal flap. Subsequently, the abdominal flap is carefully dissected away from the umbilicus, progressing toward the xiphoid process.

After that, the patient is positioned in a semi-flexed posture. The abdominal flap is temporarily closed, predominantly at its center, using staples. This closure aids in assessing the amount of excess tissue that requires excision and facilitates planning for the new position of the umbilicus. The new position is determined by palpating beneath the abdominal flap to locate the umbilicus, and a mark is placed accordingly on the abdomen. After that, if divarication of the recti is present, plication of the anterior rectus sheath is done. Then, two drains are inserted in the abdomen and fixed using Alsherawi technique [5]. Subsequently, fixation of the umbilicus is performed.

Technique 1 (performed by Dr. A. Alsherawi)

**Figure 1:**
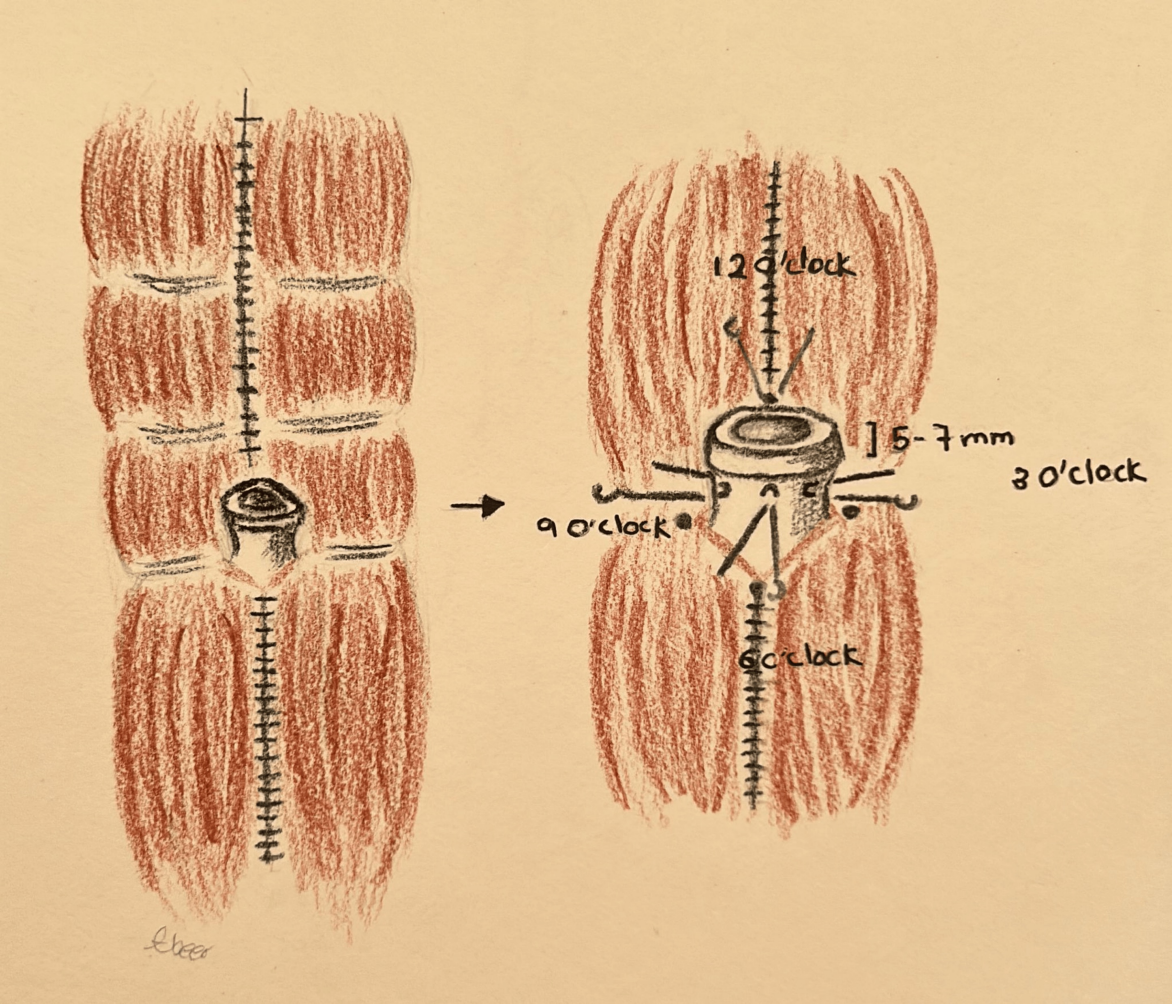
The illustration shows the position of the sutures and the distance between them and the dermo-epidermal junction of the umbilicus in technique 1 Drawings by Dr. Abeer Alsherawi

The umbilical skin is further excised and tailored to achieve the final shape and size. A diamond-shaped incision is made at the pre-marked new umbilical position on the abdominal flap, followed by dissection through the subcutaneous fat until the umbilicus is reached. The area surrounding the diamond incision (approximately 1-2 cm) is defatted to create a depth around the umbilicus, facilitating the placement of sutures in concealed areas. The umbilical stalk is anchored to the fascia at four points, approximately 5-7 mm proximal to the dermo-epidermal junction of the umbilicus. These points are located at 12, 6, 3, and 9 o’clock positions (Figure 1). Closure of the umbilical wound is performed in two layers: deep dermal inverted sutures using Monocryl 3-0, followed by skin closure with simple interrupted sutures using either 4-0 Monocryl or 5-0 Ethilon (Video 1).

**Video 1:** Surgical illustration of the first technique

Technique 2 (performed by Dr. S. Alharami)

**Figure 2:**
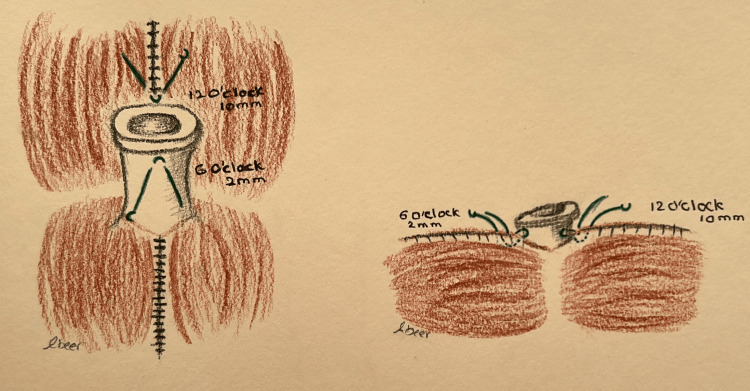
The illustration shows the position of the sutures taken to fix the umbilical stalk in technique 2 Drawings by Dr. Abeer Alsherawi

**Figure 3:**
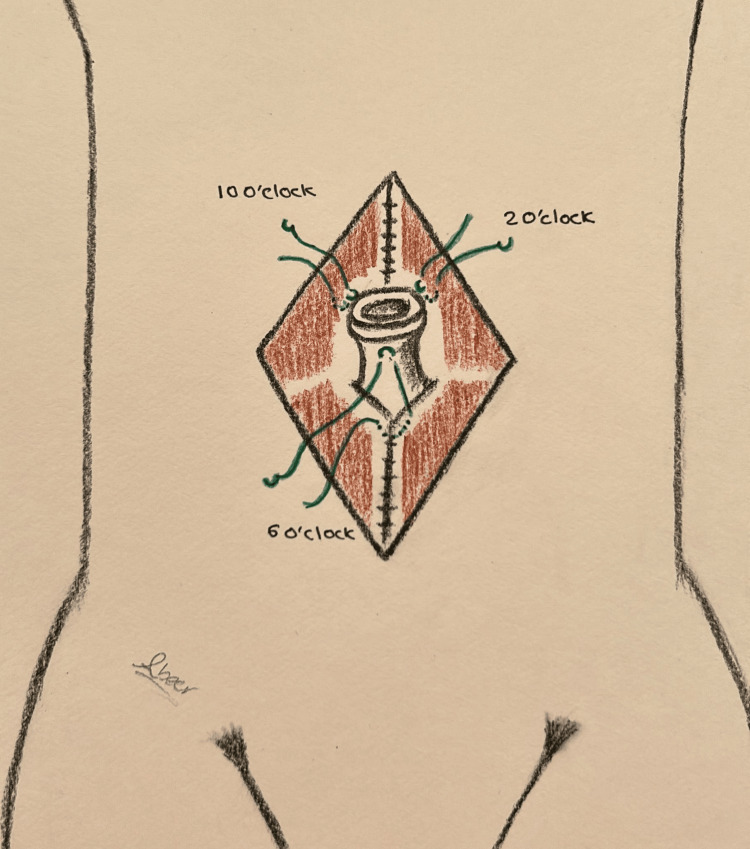
The illustration shows the position of the three main sutures taken to fix the umbilicus to the fascia in technique 2 Drawings by Dr. Abeer Alsherawi

**Figure 4:**
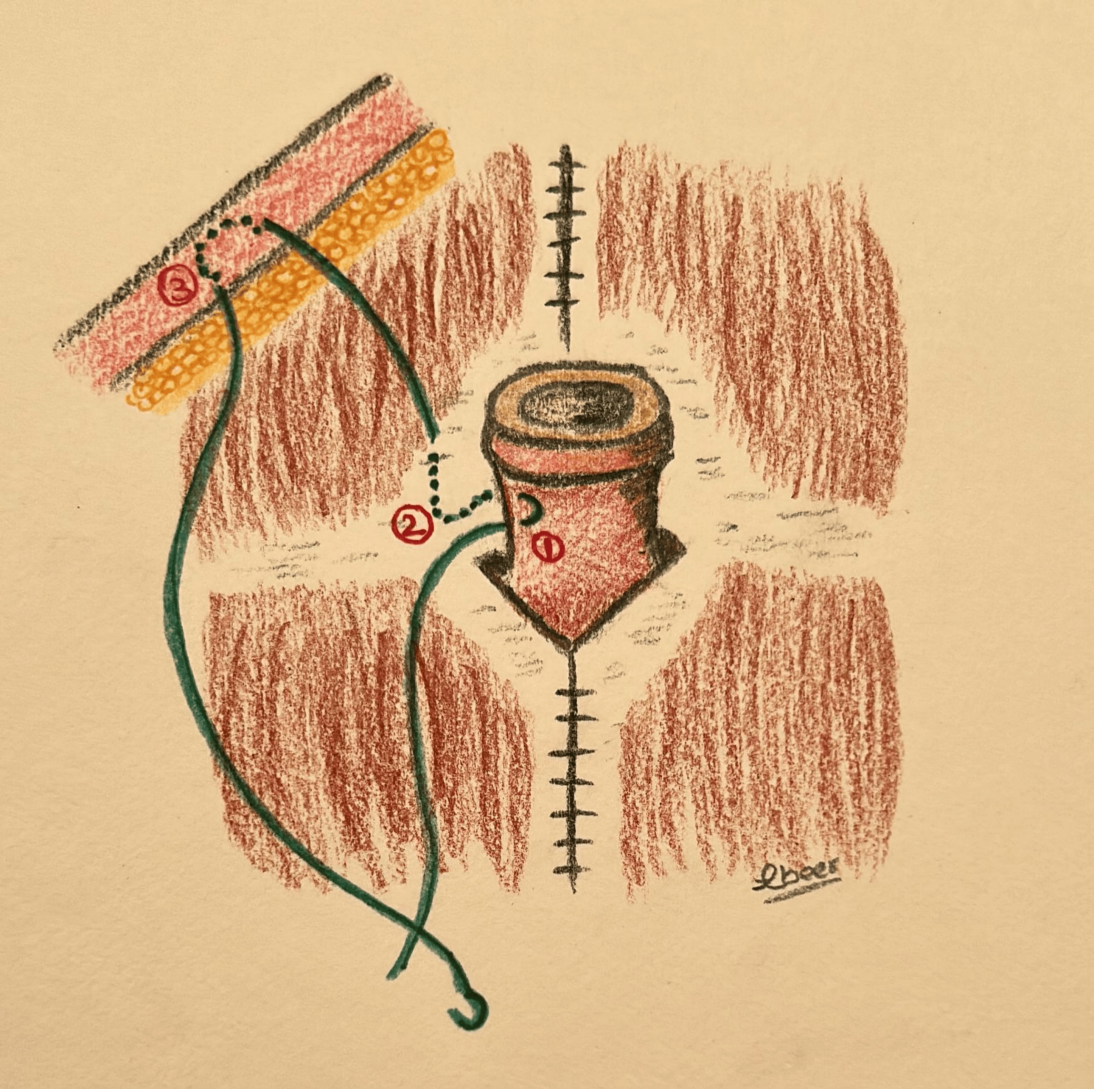
The illustration shows the direction of the three main sutures taken to fix the umbilicus and the abdominal flap to the fascia in technique 2: 1: umbilicus dermis; 2: rectus fascia; 3: abdominal flap dermis Drawings by Dr. Abeer Alsherawi.

The umbilical skin is further excised and shaped to achieve the desired shape and size. A diamond-shaped incision is made at the pre-marked new umbilical position on the abdominal flap, followed by dissection through the subcutaneous fat until the umbilicus is reached (Figure 3). The area surrounding the diamond incision (approximately 1-2 cm) is defatted to create depth around the umbilicus, facilitating the placement of sutures in concealed areas. The umbilical stalk is anchored to the fascia at the 12 o'clock position, approximately 10 mm proximal to the dermo-epidermal junction, and at the 6 o'clock position, around 2 mm proximal to the dermo-epidermal junction (Figure 2). Three main sutures using Vicryl 3-0 are placed at the 10, 2, and 6 o'clock positions to secure the umbilicus in its final position. Each suture is single-threaded with its needle, passing from the dermis to the fascia, and then secured using artery forceps. Each suture is then passed through the dermis of the abdominal flap (diamond opening) and tied with the other end, ensuring the approximation of both abdominal and umbilical skin layers above the fascia immediately (Figure 4). Closure of the umbilical wound is performed in two layers: deep dermal inverted sutures using Vicryl 3-0, followed by skin closure with simple interrupted sutures using either 4-0 Monocryl or 5-0 Ethilon (Video 2).

**Video 2:** Surgical illustration of the second technique

**Figure 5:**
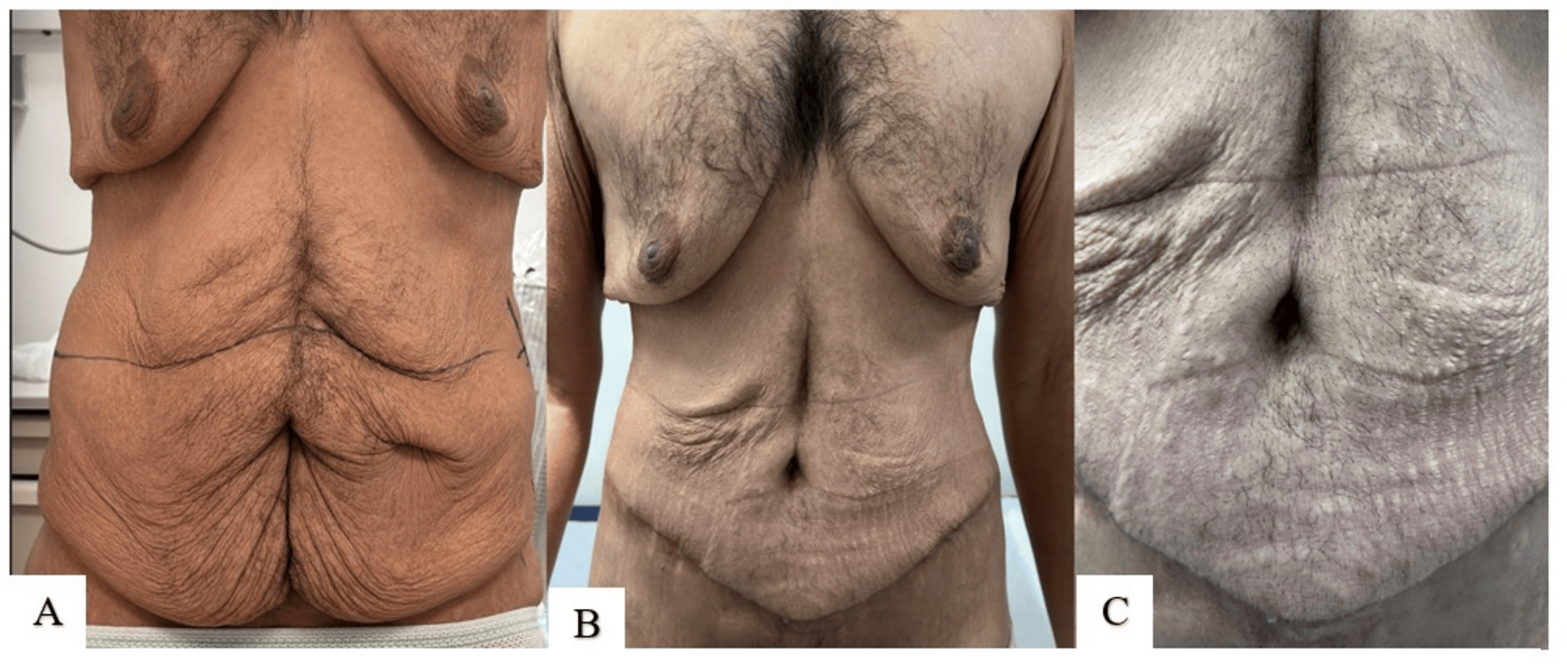
Technique 1 (A. Alsherawi): preoperative photo (A); postoperative results at six weeks (B, C)

**Figure 6:**
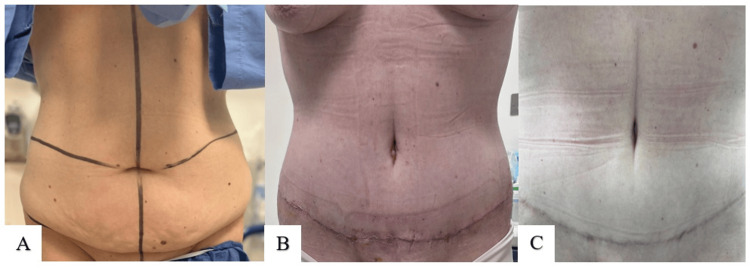
Technique 1 (A. Alsherawi): preoperative photo (A); postoperative result at two weeks (B); postoperative result at six months (C)

**Figure 7:**
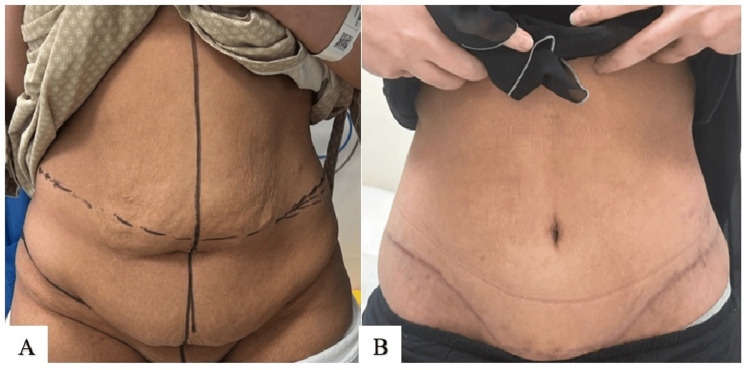
Technique 1 (A. Alsherawi): preoperative photo (A); postoperative result at three months (B)

The results of the first technique are demonstrated in Figures 5-7.

**Figure 8:**
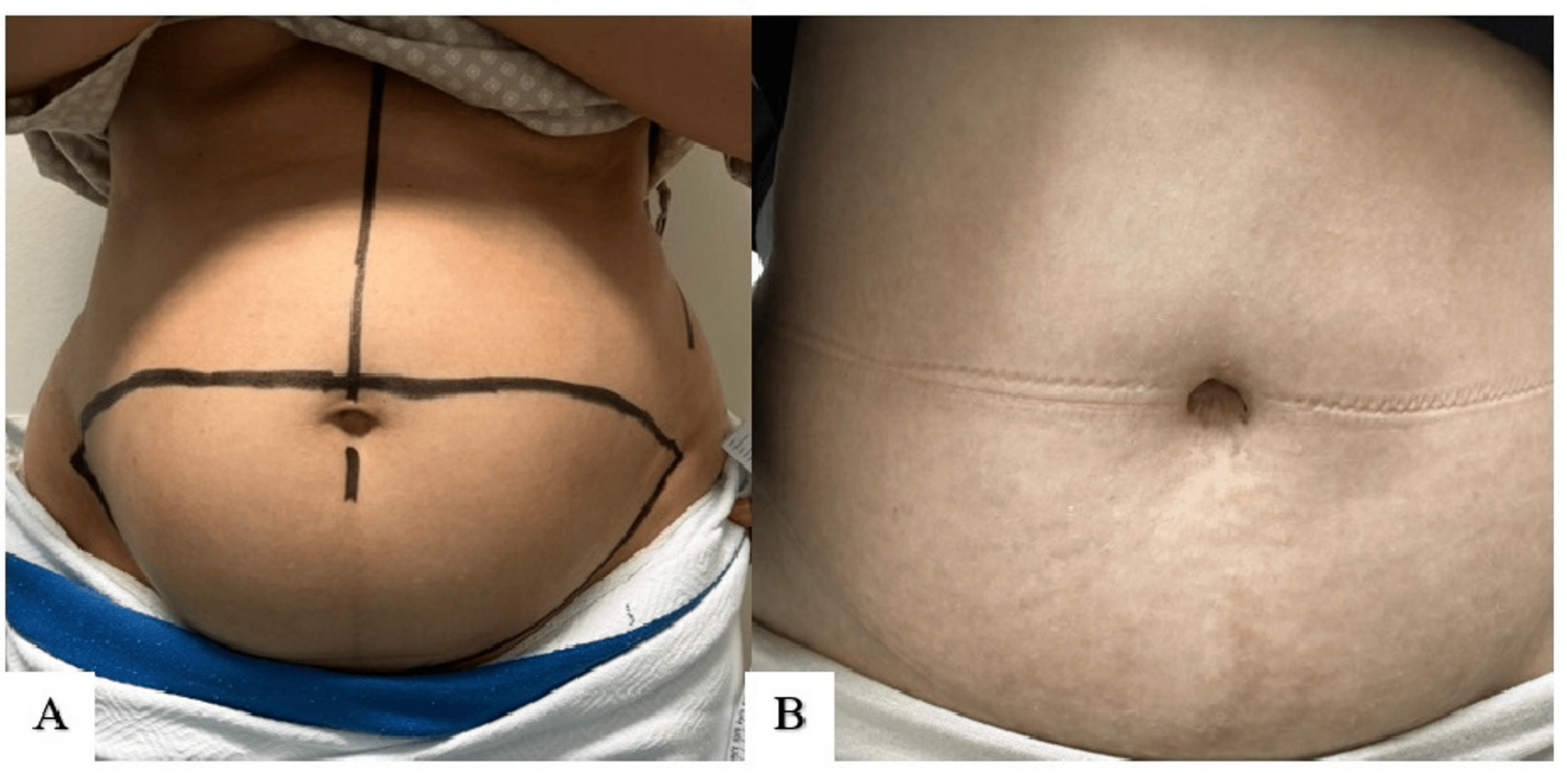
Technique 2 (S. Alharami): preoperative photo (A); postoperative result at six months (B)

**Figure 9:**
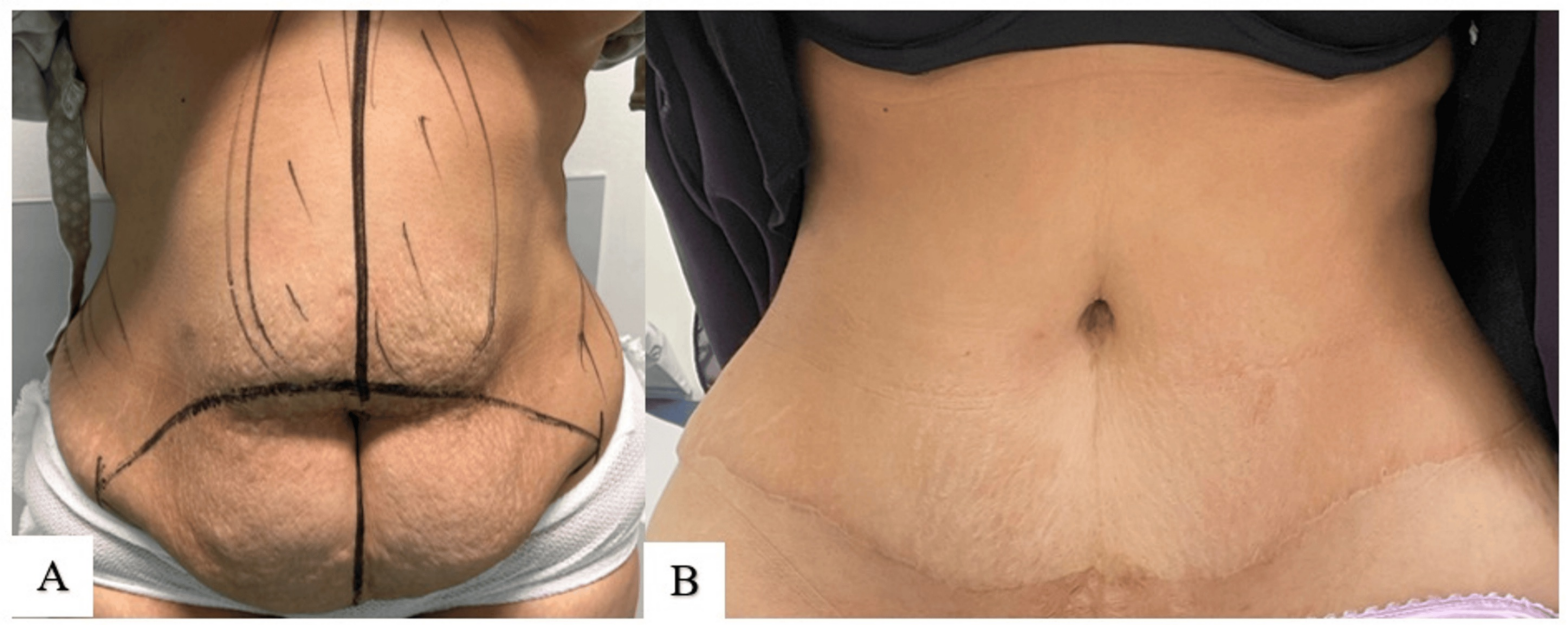
Technique 2 (S. Alharami): preoperative photo (A); postoperative result at 2.5 years (B)

**Figure 10:**
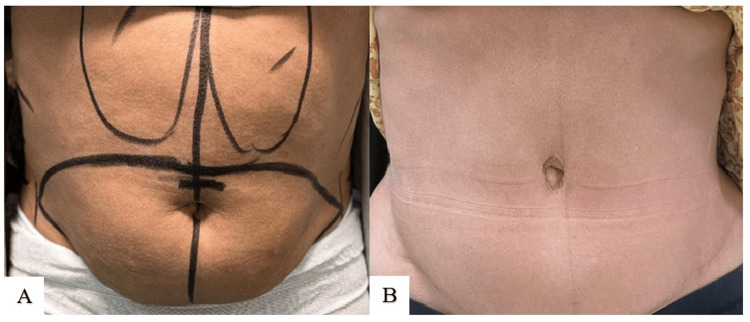
Technique 2 (S. Alharami): preoperative photo (A); postoperative result at 2.5 years (B)

The results of the second technique are demonstrated in Figures 8-10.

## Discussion

Several umbilicoplasty techniques have been introduced over the past years. However, most of the umbilicoplasty methods seek the same outcome, which is a naturally appearing umbilicus. The term "naturally appearing" refers to a small, oval umbilicus with a superior hood [1]. Another important aim of umbilicoplasty is the concealment of scars. This was highlighted by Bruekers et al. by presenting an “unscared” umbilicoplasty method that resulted in a high patient satisfaction rate [6]. This paper discusses two umbilicoplasty techniques and their respective advantages.

The key steps in the first umbilicoplasty technique involve attaching the umbilical stalk at four points at 12, 3, 6, and 9 o’clock, which are all about 5-7 mm proximal to the dermo-epidermal junction of the umbilicus. In addition, it involves debulking the fat around the diamond-shaped incision along with the closure of the wound in two layers. These steps are performed to enable natural contouring to occur between the abdomen and umbilical edges, ensuring that the new umbilicus has depth and that the scar is hidden. Yazar et al. have also mentioned attaching the umbilical stalk at four points to create depth and prevent the protrusion of the umbilicus, which was proven to be successful [2]. Dogan et al. have focused on concealing umbilicoplasty scars rather than creating depth by adopting a “cut as you go method”, which involves shortening the umbilical island to 4-8 mm [7]. This allows the scaring tissue to be centralized instead of having a circular scar around the umbilicus. Furthermore, the defatting of surrounding abdominal tissue is also practiced by Lesavoy et al. to attain an embedded appearance of the umbilicus within the anterior abdominal wall [8].

The second technique used at our facility involves fixing the umbilical stalk to the fascia at 12 and 6 o'clock with varying depths. The 12 o’clock point is attached at 10 mm, while the 6 o’clock point is attached at 2 mm proximal to the dermo-epidermal junction. Also, the umbilicus is secured with three main sutures at 10, 2, and 6 o'clock, connecting the umbilical dermis, rectus fascia, and flap dermis. These steps help maintain the depth of the umbilicus, ensure the formation of a superior hood, and prevent the occurrence of umbilical stenosis. Another innovative method of preventing umbilical stenosis is discussed by Kachare et al.; they performed inverted U-shaped umbilicoplasty followed by the insertion of an earplug into the umbilicus for six to eight weeks postoperatively [9]. Alternatively, a paper comparing inverted-U vs. vertical oval incision umbilicoplasty reported that the superior hood is emphasized by anchoring the umbilicus to the fascia at 2, 10, and 12 o’clock [10]. Of note, although the appearance of umbilical hooding might provide a natural and youthful look to the abdomen, it could result in visible scars. Furthermore, postoperative weight changes, quality of skin, and gravity can affect the long-term appearance of the superior hood [11].

Although surgeons aim to achieve the best results initially, complications may still arise, necessitating revision umbilicoplasty in a separate setting. Therefore, detailed surgical consent should be obtained, explaining all possible consequences [12]. The two umbilicoplasty techniques performed at our center have consistently resulted in high patient satisfaction. They are both reliable methods suitable for all types of body habitus. Additionally, they ensure scar concealment, provide an aesthetically pleasing look, and have a low risk of complications.

## Conclusions

In this report, we describe two uncomplicated, smooth, and safe modifications to the umbilicoplasty technique. These modifications are easy to implement, capable of securing the umbilicus in its position for a long period, and do not pose any risks related to the skin. In the future, we aim to conduct a comparative study assessing the long-term outcomes of both techniques.
